# The role of petal transpiration in floral humidity generation

**DOI:** 10.1007/s00425-022-03864-9

**Published:** 2022-03-04

**Authors:** Michael J. M. Harrap, Sean A. Rands

**Affiliations:** 1 School of Biological Sciences, University of Bristol, Bristol, BS8 1TQ UK; 2 Present Address: The John Krebs Field Station, University of Oxford, Wytham, Oxford, OX2 8QJ UK

**Keywords:** Angiosperm, Floral evolution, Floral traits, Pollinator cue, Robot arm

## Abstract

**Main conclusion:**

Using petrolatum gel as an antitranspirant on the flowers of California poppy and giant bindweed, we show that transpiration provides a large contribution to floral humidity generation.

**Abstract:**

Floral humidity, an area of elevated humidity in the headspace of flowers, is believed to be produced predominantly through a combination of evaporation of liquid nectar and transpirational water loss from the flower. However, the role of transpiration in floral humidity generation has not been directly tested and is largely inferred by continued humidity production when nectar is removed from flowers. We test whether transpiration contributes to the floral humidity generation of two species previously identified to produce elevated floral humidity, *Calystegia silvatica* and *Eschscholzia californica*. Floral humidity production of flowers that underwent an antitranspirant treatment, petrolatum gel which blocks transpiration from treated tissues, is compared to flowers that did not receive such treatments. Gel treatments reduced floral humidity production to approximately a third of that produced by untreated flowers in *C. silvatica*, and half of that in *E. californica*. This confirms the previously untested inferences that transpiration has a large contribution to floral humidity generation and that this contribution may vary between species.

**Supplementary Information:**

The online version contains supplementary material available at 10.1007/s00425-022-03864-9.

## Introduction

Floral humidity, an area of elevated humidity in the headspace of the flower (relative to the environment), has been detected in several flower species across different families (Harrap et al. 2020a). Flower species have been found to vary in the intensity of floral humidity produced (the difference in humidity between the floral headspace and the environment) and in the humidity structure (the shape and location of elevated humidity within the flower headspace). Floral humidity may have important influences on flower function and fitness. Floral displays are multimodal, and produce many different kinds of signals and cues simultaneously, such as visual (Dyer and Chittka 2004; Raine and Chittka 2008; Foster et al. 2014; Muth et al. 2015), olfactory (Kunze and Gumbert 2001; Wright and Schiestl 2009; Lawson et al. 2018), texture (Whitney et al. 2009, 2011), temperature (Dyer et al. 2006; Whitney et al. 2008; Harrap et al. 2017, 2020b), and electrostatic cues (Clarke et al. 2013). Pollinators use different floral signalling modalities to inform foraging decisions (Raguso 2004; Leonard et al. 2011, 2012; Leonard and Masek 2014). Floral humidity, as part of this multimodal floral display, can influence pollinator foraging decisions. Hawkmoths (von Arx et al. 2012), bumblebees (Harrap et al. 2021), and flies (Nordström et al. 2017) have innate preferences for flowers that produce higher floral humidity intensities when they are able to choose between flowers producing differing intensities. Furthermore, floral humidity differences between flowers, regardless of whether elevated rewards are associated with higher floral humidity production or not, can aid bumblebee learning of rewarding flowers (Harrap et al. 2021). By influencing floral preferences and learning, floral humidity may affect foraging success of naïve and experienced pollinators (Raine and Chittka 2008) and will in turn influence visitation rates of pollinators and thus pollen receipt and export (Ashman et al. 2004; Schiestl and Johnson 2013). Humidity production by flowers, perhaps alongside production from vegetative tissue, may also affect patch-level foraging decisions of pollinators, allowing them to locate areas of elevated foliage (where suitable forage is more likely to be) resulting in similar impacts on pollinator and plant fitness (Wolfin et al. 2018). Floral humidity may have further influences on plant fitness that have not yet been directly investigated. Humidity conditions influence pollen water content, which in turn influences the viability and germination ability of pollen (Nepi et al. 2001; Hase et al. 2006). Floral humidity may therefore have an influence on pollen viability, perhaps maintaining an environment more suitable for pollen.

Floral humidity is believed to be generated through a combination of evaporation of liquid nectar of flowers and release of water vapour from the flower via transpiration, particularly from flower petals. However, other floral characteristics, such as floral structure, influence how humidity produced accumulates in the flower headspace (von Arx et al. 2012; Harrap et al. 2020a). How flowers generate humidity has received little investigation in the previous research, and what research has been conducted on floral humidity generation has focused on the role of nectar evaporation. Removal of nectar from flowers of evening primrose *Oenothera caespitosa* resulted in a decrease in floral humidity production, and blocking nectaries also yielded similar results (von Arx et al. 2012), confirming the role of nectar evaporation in floral humidity generation. This is also supported by evidence that, within flower headspaces, proximity to nectaries is associated with increased humidity (Corbet et al., 1979a, b; von Arx et al. 2012; Harrap et al. 2020a). Evidence of the contribution of transpiration to floral humidity is comparatively limited, and largely inferred. That nectar removal did not completely reduce floral humidity production in *O. caespitosa* indicates that there is an additional humidity source contributing to floral humidity (Harrap et al. 2020a), and the most likely candidate for this is floral transpiration. Similarly, humidity detected from *O. caespitosa* was reduced when petal surfaces, but not nectar or nectaries, were shielded from sensors (von Arx et al. 2012), indicating that petals contribute to floral humidity, likely via transpiration. However, there are other potential sources of floral humidity generation, such as the moist surfaces of reproductive structures. These may explain floral humidity production independent of nectar evaporation. Although the inference of transpiration’s role in floral humidity generation is logical, the contribution of transpiration to floral humidity generation has not been tested.

Petal permeability, and therefore floral transpiration rates, can be influenced by cuticle thickness, surface area, and chemical composition (Hajibagheri et al. 1983; Buschhaus et al. 2015; Guo et al. 2017; Cheng et al. 2019). The presence, density, and activity of petal stomata also contributes to rates of floral transpiration (Hew et al. 1980; van Doorn 1997; von Arx et al. 2012; Huang et al. 2018). Confirmation of the role of transpiration in floral humidity generation will confirm whether differences in such traits may help explain the diversity of floral humidity seen in angiosperms (Harrap et al. 2020a). Environmental conditions (Gates 1968; Rawson et al. 1977; Jolliet and Bailey 1992; Schreiber 2001) and plant daily cycles can affect transpiration (Simon et al. 2020), including floral transpiration cycles (Azad et al. 2007; Lü et al. 2011; Huang et al. 2018), so the extent to which floral humidity depends on transpiration may indicate potential for parallel changes in floral humidity with time and conditions, perhaps leading to changes in humidity cues. Furthermore, as floral humidity produced by transpiration is not affected by nectar removal (von Arx et al. 2012), the role of transpiration in different species (particularly relative to nectar evaporation) may influence how well floral humidity cues indicate the temporary rewardlessness of the flower due to recent visits, influencing the extent that floral humidity can function as a directly ‘honest signal’ for pollinators (von Arx 2013). Understanding the influence that floral transpiration has on floral humidity is therefore important for understanding both the function and evolution of floral humidity.

In this study, we demonstrate the contribution of petal transpiration to floral humidity generation in two flower species previously identified to produce elevated floral humidity. A simple antitranspirant treatment, petrolatum gel, was applied to the petals of flowers, serving to block transpirational water loss from petals. Humidity in the flower headspace was then measured using robotic sampling techniques and compared between treated flowers and those of the same species which had not undergone this treatment. In this way, we were able to evaluate floral humidity production with (untreated flowers) or without (gel-treated flowers) the contribution of petal transpiration.

## Materials and methods

### Collection and preparation of samples

The role of transpiration in floral humidity generation was tested in the flowers of giant bindweed *Calystegia silvatica* (Kit.) Griseb. and California poppy *Eschscholzia californica* Cham.. These species are appropriate choices for demonstrating the role of transpiration in floral humidity generation, as both produce larger amounts of floral humidity compared to other flower species (Harrap et al. 2020a) comparable to levels which have been demonstrated to be able to influence pollinator foraging decisions (von Arx et al. 2012; von Arx 2013; Wolfin et al. 2018; Harrap et al. 2021). *E. californica* has been identified (along with other poppies) to produce little in the way of nectar rewards (Hicks et al. 2016), and so nectar evaporation is unlikely to explain *E. californica* humidity production. *C. silvatica* produces more substantial nectar rewards (Baude et al. 2016), but is a useful study species as the Convolvulaceae are known to conduct large amounts of petal transpiration (Patiño and Grace 2002).

Humidity sampling and treatments were also carried out on leaves of common ivy *Hedera helix* L. Ivy leaves lack extrafloral nectaries or similar secretions (Vezza et al. 2006), so humidity produced by the leaf should be solely from transpiration sources via leaf stomata or epidermal tissue (von Arx et al. 2012). Consequently, leaf samples serve as a positive control for our gel treatment, confirming the extent that gel treatments block plant transpiration.

All plant material was collected from sites within walking distance of the University of Bristol main campus (51.45 N 2.60 W). Flowers were cut on the stem, so no leaf remained on the cutting, but sepals were retained, and individual ivy leaves were cut with the majority of the petiole remaining attached. Immediately after cutting, the stems attached to samples were stuck through a hole in the cap of a 24 cm^3^ plastic horticulture tube, prefilled with water to within 2 cm of the lid. Samples were transported upright in a closed cardboard box. Flowers and leaves were only collected in dry conditions, so that no standing water from condensation or rain was present on flowers to influence the floral humidity measurements. Flowers and leaves were only used if they were fully open and did not show signs of age, disease, or damage.

Samples were collected to allow two samples (two flowers or two leaves) to be presented to the robot in pairs (spares were also collected to accommodate any breakage during treatment application). When selecting flowers and leaves that would be presented for sampling as ‘Gel’ and ‘Untreated’ sampling pairs (see below for treatment details), care was taken to collect flowers of approximately the same size, measured using a ruler across either the horizontal span of a flower, or the width of the leaf’s widest point. To control for size effects, flowers and leaves presented as sampling pairs of one ‘Gel’ and one ‘Untreated’ sample were within 10 mm of their partner’s span. For sampled pairs of ‘Unhandled’ treatments, size was allowed to vary to capture as wide a range of the diversity as possible. Flowers and leaves presented for sampling in the same pair were always collected from the same site, so that flowers had grown in comparable conditions prior to being collected.

**Fig. 1:**
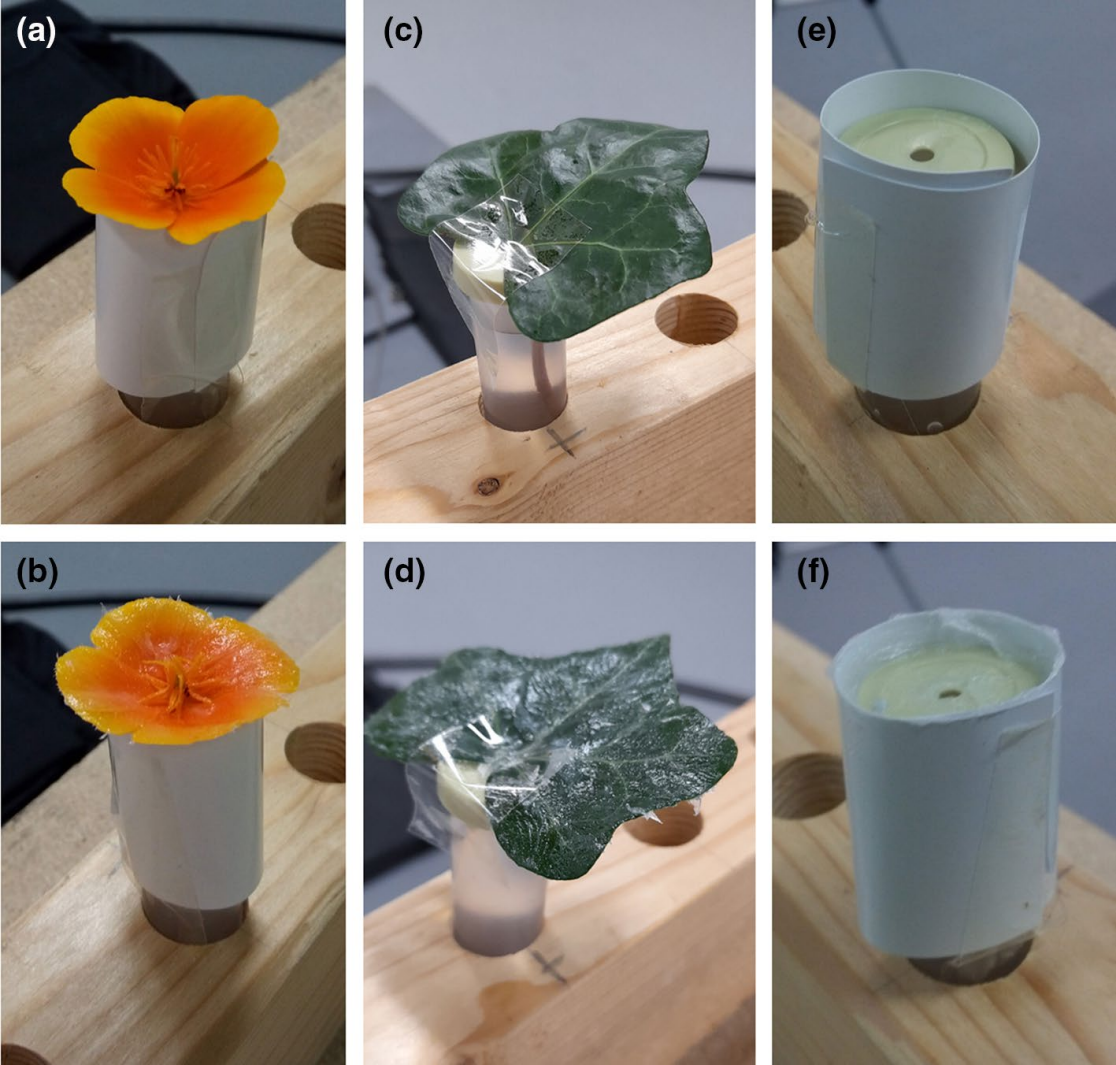
Prepared samples of *Eschscholzia californica* flowers (**a**, **b**), *Hedera helix* leaves (**c**, **d**), and tube control samples (**e**, **f**). **a**, **c**, **e** ‘Untreated’ samples, where samples were handled as if gel were applied. **b**, **d**, **f** ‘Gel’ treatment where gel was applied to sample surfaces. These samples are within a tube rack on a table before the robot, as they would be presented for humidity sampling

Prior to measurement, flowers were fixed in position, orientated vertically upwards, to prevent the measuring points of humidity transect differing with respect to the flower’s location due to the flower moving. Under natural conditions, both species have vertically orientated flowers, although *C. silvatica* can have differing flower orientations dependent on where it grows. Thus, fixing the orientation of the flowers is unlikely to negatively affect the flowers. Flowers were fixed in position with a cylinder of rigid paper-card (Professional laser printer paper, Hewlett-Packard, Startbaan, The Netherlands) fixed with tape (Scotch, St. Pauls, MN, USA) to the outside of the horticultural tube, so the flowers rested flat facing vertically upwards (Fig. 1a and b). The size of the cylinder required varied with the individual flower and species being sampled, but normally protruded only a few centimetres above the tube’s lid and was of a comparable width to the tube itself. Care was taken not to affix this cylinder, so that it constrained or compressed the flower.

Leaf samples could not be supported and orientated by card cylinders, and instead were secured to the horticultural tube with a section of tape after treatment application (described below). This tape was placed over the base of the leaf and the hole in the horticultural tube’s lid and continued down the side of the tube (Fig. 1c and d). This secured the leaves, which were angled slightly upwards relative to the horticultural tube cap. The irregular curving and shape of leaves meant that they did not always present a level surface, but this was accounted for when setting the ‘transect central point’ (see below).

### Preparation of control samples

Two further ‘control samples’ were conducted. These were the ‘Dry tube’ control, where a card support cylinder was attached around an empty horticultural tube (protruding 1 cm above the lid), and the ‘Full tube’ control which was the same but with the tube filled with water to within 2 cm of the lid, similar to leaf and flower samples (Fig. 1e and f). Controls served two purposes. First, when untreated, they allowed us to assess the extent that humidity differences detected between the focal and background probe are due to sources extraneous to the leaves or flowers placed within the tube. Evaporation of water via the hole in the lid could contribute to humidity differences (which we could measure with the ‘Full tube’ control). Similarly, random mixing of the air in the room can lead to uneven humidity environments and differences in humidity between the probes due to them differing in position (which could be measured with the ‘Dry tube’ control). Second, it is possible impurities in the petrolatum applied to samples evaporates or absorbs moisture allowing gel to affect humidity independently of blocking transpiration. By comparing the effect of gel treatments on the controls, we could evaluate whether the gel itself influences humidity.

### Antitranspirant treatments

Three treatments were conducted across flower, leaf, and control samples. Flower samples underwent either the ‘Gel’, ‘Untreated’, or ‘Unhandled’ treatments (Table 1). Leaf and control samples underwent only the ‘Gel’ and ‘Untreated’ treatments.

**Table 1:** A summary of the treatments applied to samples and the numbers of individuals of each sample type subjected to each treatment

Treatment	Description	*Calystegia silvatica*	*Eschscholzia californica*	*Hedera helix*	Dry Tube Control	Full Tube Control
Gel	Petrolatum gel is applied by hand over the abaxial and adaxial surface of flower petals and sepals and leaves, or the top of the tube lid in controls	25 (7)	25 (6)	8	15 (2)	19
Untreated	Petrolatum gel is not applied. Samples are handled with dry hands as if gel were applied	25 (7)	25 (6)	8	15 (2)	19
Unhandled	Petrolatum gel is not applied. Samples (flowers only) are not handled beyond collection and fixing of position in preparation for robot sampling	20 (6)	20 (6)	–	–	–

Where ‘–’ is given, samples were not subjected to that treatment. Bracketed values indicate the number of samples within that treatment that were also monitored for temperature differences

Samples receiving the ‘Gel’ treatment had a simple antitranspirant applied to their surface. In flowers receiving the ‘Gel’ treatment, petrolatum gel (‘Boots baby petroleum jelly’, Boots, Nottingham, UK) was applied evenly by hand over the abaxial and adaxial surface of petals, and over the flower sepals (Fig. 1b). Gel was not applied to floral reproductive structures. About the base of the petals’ upward (adaxial) surface where nectaries and reproductive structures were present, gel was applied as close as possible to these structures without covering them, meaning that small areas of the petal near these structures remained untreated. This was to avoid the chance gel treatment may remove unconsidered contributions to floral humidity aside from petal (and sepal) transpiration such as nectar evaporation or release of moisture from reproductive structures. Similarly, in leaf samples that underwent the ‘Gel’ treatment, adaxial and abaxial surfaces of the leaf were covered with petrolatum gel (Fig. 1c), and in tube control samples, gel was applied over the horticultural tube lid and around the protruding section of the inside of the card cylinder (Fig. 1f). Samples that underwent the ‘Untreated’ treatment had no antitranspirant gel applied on them, but the samples were handled as if it were being applied. Samples were handled with dry hands as described above, with similar spreading motions across the relevant surfaces associate with application of the gel. Only flower samples underwent the ‘Unhandled’ treatment, where no further handling of flowers was conducted following picking and fitting of card cylinder supports. Flowers in the ‘Unhandled’ treatment, being otherwise unmanipulated, allowed further evaluation of ‘normal’ floral humidity production. Comparing humidity production between the ‘Untreated’ and ‘Unhandled’ treatments allowed us to account for the influences of the handling procedures involved with application of the gel antitransiprant when assessing the humidity production of treated flowers. Comparing ‘Gel’ treated to ‘Untreated’ flowers allowed assessment of antitranspirant treatments on humidity production, accounting for the effects of handling. If at any time a sample broke during treatment application, it was replaced.

The humidity sampling sequence was conducted on a pair of samples of the same type (i.e., a pair of leaves or similar flowers, Dry tube controls, or Full tube controls). The pair of samples presented to the robot were prepared, so that they were either: a sample that had undergone the ‘Gel’ treatment and sample that had undergone the ‘Untreated’ treatment; or two flower samples that had undergone the ‘Unhandled’ treatment. This allowed us to balance our comparisons with respect to time of sampling and any other environmental changes in the sampling area that might influence humidity production.

### Robot sampling procedure

Humidity within sample headspace (the headspaces of flower, leaf, and tube control samples) was measured using a modified version of the robot humidity transect method used in Harrap et al. (2020a, 2021); unless stated otherwise, all aspects of the humidity transects conducted were as described in those publications.

**Fig. 2:**
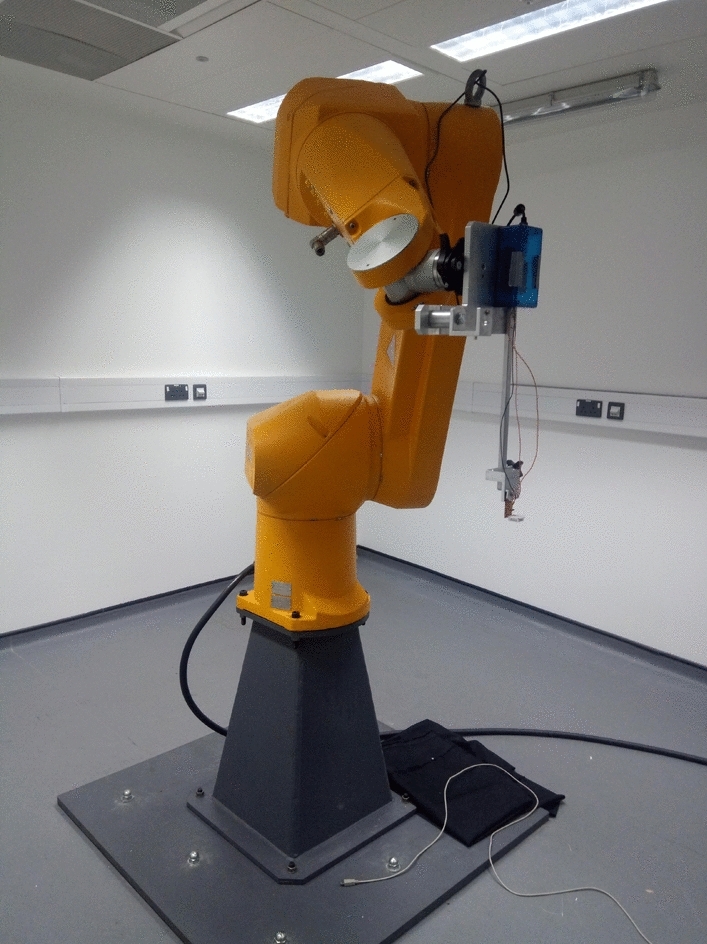
The robot arm and lab space used for headspace humidity sampling. Of note is the focal humidity probe on a 30 mm bar mounted to the end of the robot arm. Samples and the background probe would be placed on a table below the robot. For further information of robot setup, see Harrap et al. (2020a)

Humidity sampling was conducted by a Staubli RX 160 robot arm (Pfäffikon, Switzerland). Here, background humidity was measured by a humidity probe (DHT-22 humidity probe, Aosong Electronics, Huangpu, China) placed within the lab space, the ‘background’ probe. Sample headspace humidity measurements were taken with an identical probe mounted to the robot arm, the ‘focal’ probe (Fig. 2). The amount of water vapour indicated by a given relative humidity value is dependent on air temperature, with the amount of water vapour indicated by a given relative humidity value approximately doubling with a 10ºC rise in temperature (Tichy and Kallina 2014). Consequently, if the temperature varies between humidity measurements, relative humidity values cannot be compared directly, due to the amount of vapour indicated by a percent relative humidity unit differing. In such circumstances, a conversion of relative humidity measurements to absolute humidity (the mass of water vapour per volume of air) would be necessary to compare humidity between measurements. For this reason, background temperature of the lab where sampling took place was regulated. The background temperature during sampling measurements (as measured by the ‘background probe’ during sample measurements) was 22.41ºC ± 0.50 (mean ± SD). This constant lab temperature allowed comparison of relative humidity values across all sample measurements, as each percent relative humidity unit here represents a similar amount of water vapour. Due to this, while conversion to absolute humidity remains possible, this would have minimal effects on the shape of humidity profiles and the relative intensities of humidity produced between different samples and treatments. Thus, performing such a conversion would have no effect on the conclusions of the study. It was therefore deemed unnecessary to convert the relative humidity data into absolute humidity. Furthermore, this stable background temperature and use of relative humidity as our measurement of floral humidity at a given point allowed comparison with floral humidity measurements conducted previously (Corbet et al. 1979a; von Arx et al. 2012; Nordström et al. 2017; Harrap et al. 2020a, 2021), which also use relative humidity to assess floral humidity under comparable environmental conditions to those in the current study. Background relative humidity during sampling (as measured by the ‘background probe’ during sample measurements) was 54.54% ± 11.00 (mean ± SD).

**Fig. 3:**
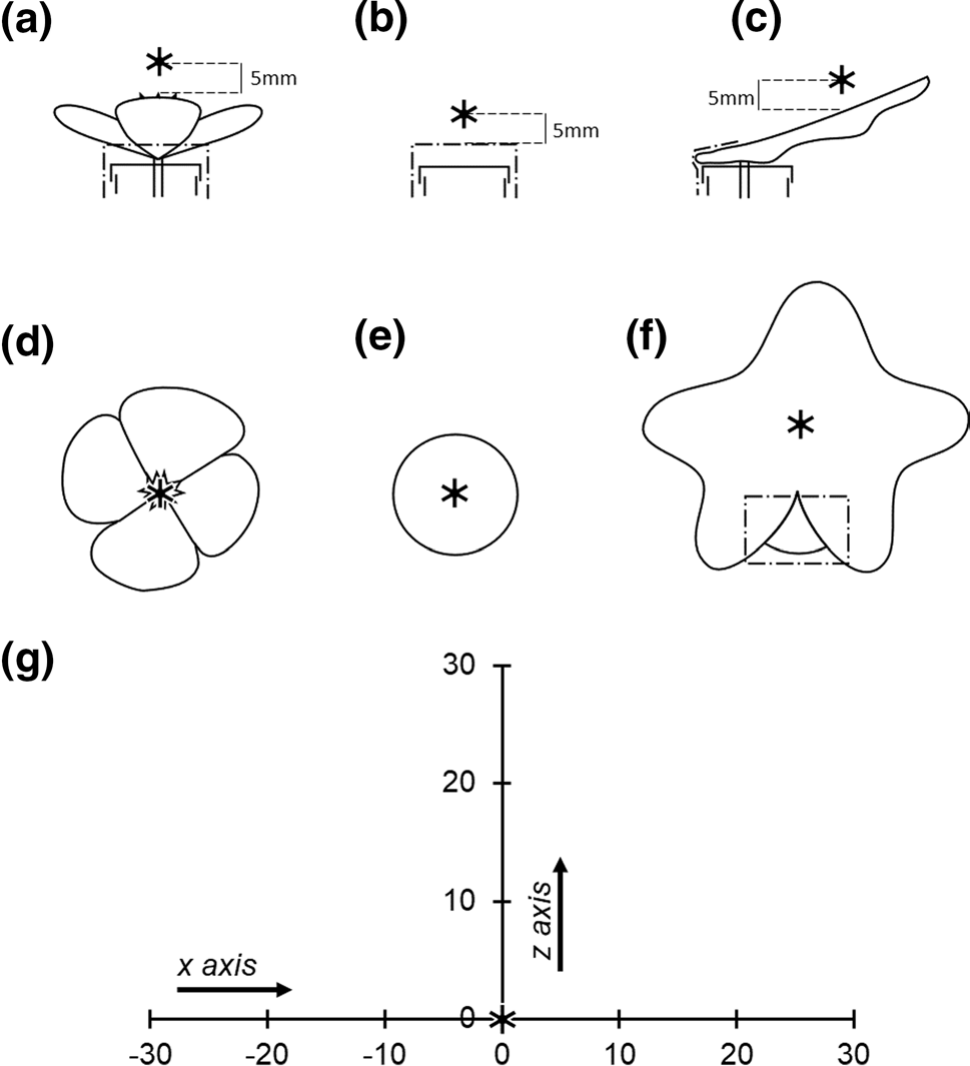
Spatial layout of humidity transects and measurement points within them relative to samples. Throughout all diagrams, the ‘transect central point’ is represented by the bold asterisk ‘*’. **a–c** The location of the transect central point relative to a flower (*Eschscholzia californica*), tube and leaf (*Hedera helix*) sample, respectively, when viewed down the horizontal* x*-axis transect. **d–f** The location of the transect central point relative to a flower, tube, and leaf sample, respectively, when viewed down the vertical *z*-axis transect. In each of these diagrams, the robot would move towards the viewer while conducting the respective transects. Dot-dash lines indicate paper and tape supports of samples. **g** The spatial layout of the humidity headspace measured above a sample, when viewed in cross section sideways on. Each measurement point is marked with a dash and described by the offset distance along that transect (in millimetres) relative to the transect central point (*x* = 0 and *z* = 0). Arrows indicate the directions the probe travels during each transect

During sampling, the robot moved the focal probe through the headspace of a sample in a set sequence of transects, conducting first an *x*-axis transect (horizontal) and then a *z*-axis transect (vertical). During these two transects, the arm stops to take humidity measurements at set measurement points (Fig. 3). Following completion of *x* and *z* transects, the arm conducted a probe calibration step, allowing measurements to account for variation in humidity estimation of the same humidity levels between probes. The positions of these transects, and humidity measurement points within them for each sample were calculated by the robot in three-dimensional space relative to a manually input ‘transect central point’. This ‘transect central point’ for flower and tube control samples was set to a point in space above the centre of the flower or tube and 5 mm higher than its highest point. For leaf samples, a point in space above the centre of its horizontal span and 5 mm higher than the highest point on this horizontal span was set as the transect central point (Fig. 3). Pairs of samples (prepared as described above) were presented to the robot arm and positions of transect central points (as well as positions required for probe calibration steps) input manually into the robot’s memory. The robot’s autonomous sequence was then activated, and the lab space was vacated. All robot sampling commenced within 1 h of sample collection.

Upon activation, the arm randomly chose a sample from within a pair and then conducted *x* and then *z* transects, measuring humidity at measurement points throughout (Fig. 3), followed by a probe calibration step. This same sequence of transects and calibration was then repeated for the other sample in the pair. After measuring both paired samples, the arm repeated this sequence on both samples in the same sampling order. Robot sampling was conducted between 2019/08/08 and 2019/08/23 and between 2020/07/06 and 2020/09/28 with sampling occurring throughout the day (between 0900 and 2000). Further detail of the layout of the sampling area and the robot sampling procedure can be found in Harrap et al. (2020a) and Supplementary Information S1.

The arm stopped for 230 s at each measurement point on both transects (Fig. 3). The first 30 s of this was settling time to account for small disruptions from arm motion (see Harrap et al 2020a, b). The following 200 s was the ‘measurement period’ for that measurement point on that transect. During the measurement period, the focal probe sampled humidity continuously, and this focal humidity at the point was corrected based on the probe control step to give corrected focal humidity (*f*_corrected_), which accounted for variation between probes in humidity estimation of the same humidity levels (see Harrap et al. 2020a, b and Supplementary Information S1 for further detail). Simultaneously, the background probe measured the background humidity (*f*_background_). During 200 s, each probe could sample humidity approximately 100 times. Change in humidity, ΔRH, between the focal and background probe was then calculated as

1 $$ \Delta RH = f_{{{\text{corrected}}}} - f_{{{\text{background}}}} , $$

where a positive value of *ΔRH* indicates the focal probe has detected an increase in humidity in the flower’s headspace relative to the background humidity. This same measurement procedure of 30 s waiting time and 200 s measurement was also carried out during probe control measurements.

Although similar, the humidity sampling procedure used here differs in several ways from that in Harrap et al. (2020a, 2021). Here, we reduced the number of measurement points on the transects, the number of replicate transects the arm conducted on each individual sample, and the number of samples presented to the arm at a time. This was done to reduce the length of each transect sequence to approximately 2 h from robot activation to completion of sampling on the last replicate transect of a sample pair (previously 21 h). Cutting a plant organ can separate its hormonal controls and interfere with water uptake (van Doorn 1997; Lü et al. 2011; Huang et al. 2018), as can antitranspirant treatments (Davenport et al. 1972; Neumann 1974), leading to drying out or wilting and changes in transpiration activity, even without blocking by antitranspirants. These effects can take time to develop, with cut flowers showing normal cycles of transpiration and water uptake within the timescales of the current or previous robot sampling procedure (Lü et al. 2011; Huang et al. 2018). Regardless, the shorter sampling sequence ensured that separated flowers were fresh, were not wilting, and were functioning more normally in terms of water uptake and transpiration where treatments were not applied.

### Analysis of humidity production

Change in humidity, *ΔRH*, as measured by the robot sampling procedure, has been found to be highly consistent within each measurement period, the *c*.100 measurements made at each sampling point, on each replicate, on each sample (Harrap et al. 2020a). We therefore used the calculated the mean *ΔRH* for each measurement period and used these in our analyses.

Analysis of sample humidity production involved two steps. In the first part, the best-fitting structure of humidity for each sample type was found, using models of humidity structure that allowed humidity production to vary as required with treatments. For each sample type, a series of linear models were fitted to the data of the *x*- and *z*-axis humidity transects. These different models described different humidity structures and allowed humidity to vary with treatment. Sample identity (the identity of the individual flower, leaf, or tube) was included in all models, as a random factor influencing humidity intensity (model intercept). The most complex models allowed humidity to vary with replicate transects and show a quadratic structure in the *x*-axis and a logarithmic structure in the *z*-axis. All other models were simplified versions of these. How well these different models described humidity structure was compared using Akaike information criterion (AIC) to identify the best-fitting model of *x-* and *z*-axis humidity structure for each sample type, using the model section criteria described in Richards (2008). These models and the associated analysis are comparable to those applied to data collected previously with this method (Harrap et al. 2020a, 2021), with the additional treatment effects included in the models. The structure of these models is described in Supplementary Information S2 as well as Suppl. Tables S1 and S2.

In the second part of the analysis, the effects of treatment on humidity production in each sample type were evaluated. Here, versions of the best-fitting model of humidity structure with different treatment effects were fitted to the data of each sample type. These models differed in how humidity production changed with treatments, treatment had either: no effect on humidity production, the *T0* (*x*-axis) and *Tz0* (*z*-axis) models; an effect on humidity intensity only (model intercept), the *T1* and *Tz1* models; an effect on humidity structure only, the *T2* and *Tz2* models; an effect on both humidity intensity and structure the *T3* and *Tz3* models; or an effect on both humidity intensity and structure as well as how humidity changes with replicate effects, the *T4* and *Tz4* models. Where the best-fitting model of humidity structure for a sample, as selected above, did not include changes in humidity with replicate effects, the *T4* and *Tz4* models were not fitted to the data (as there were no replicate effects for treatment to alter). Depending on the best-performing humidity structure model, either the *T3* and *Tz3* or the *T4* and *Tz4* represent the ‘full’ model selected in the humidity structure step (it was possible, if a flat model with no replicate effects was favoured, for *T1* and *Tz1* be the ‘full’ model at this stage, this was not the case in any of our samples).

In both tube and leaf samples, there were only two treatments (‘Untreated’ and ‘Gel’), so the models described up to this point identify the nature of treatment’s effects. However, in flower samples, there were three treatments (‘Unhandled’, ‘Untreated’ and ‘Gel’). To further assess the effects of these three different treatments additional variants of the treatment effects models described above were fitted to the data that grouped together the effects of different treatments. These were identified using a subscript after the model names described above: a lack of a subscript entry indicating all treatments differ; ‘TCwP’, Untreated and Gel treatments are grouped together; ‘TCwU’, Untreated and Unhandled treatments are grouped together; ‘TPwU’, Gel and Unhandled treatments are grouped together. For example, model *T3*_*TCwP*_ describes treatment effects on both the humidity intensity and structure, but groups the ‘Gel’ and ‘Untreated’ treatments together; thus, only the ‘Unhandled’ treatment differs. The *T0* and *Tz0* models describe no treatment effects; thus, there were no further variants of these models. The structure of treatment effect models is described in Supplementary Information S2 and Suppl. Table S3.

How well these various treatment effects models described humidity structure was compared using AIC to identify the best-fitting treatment effects in *x-* and *z*-axis for each sample type, using criteria described in Richards (2008). This two-step process of selecting a humidity structure model and then a treatment effect model were favoured as it reduced the complexity of the analysis, reducing the number of models fit to the data. Following identification of the model that best described treatment effects of each sample, humidity intensity summary values ${X}_{t}^{\mathrm{max}}$ (the position of the mean peak in humidity production over the x-axis transect, relative to the transect central point) and ${\Delta RH}_{x}^{\mathrm{max}}$(the average peak in humidity production over the *x*-axis transect) were calculated according to the best-fitting treatment model for each treatment of each sample and each replicate transect when it was included in the best-fitting model. These summary values, respectively, give a conservative estimate of humidity structure symmetry and humidity intensity produced by each sample and treatment. For further detail on summary values and their calculation, see Harrap et al. (2020a) and Supplementary Information S3.

### Monitoring of sample temperature

Floral transpiration can play an important role in floral temperature regulation (Patiño and Grace 2002) so blocking transpiration may influence floral temperature. Understanding antitranspirant treatments’ influence on floral temperature may help explain floral humidity changes and the relationship between these traits. Temperature was therefore monitored alongside humidity sampling for a subset of samples (Table 1). In these instances, a thermal camera (FLIR *E60bx*, FLIR systems Inc., Wilsonville, OR, USA) was mounted on a tripod viewing the sample pair from an elevated side on angle. A small (*c*. 10 × 20 cm) aluminium foil multidirectional mirror was placed in view of the camera against with the bottom of the horticultural tube rack. This fully charged thermal camera was automated to take a thermal image upon activation just before humidity sampling began and every subsequent 15 min using the FLIR Tools software live feed functionality and autoclicker software (written within AutoHotkey) running on an attached laptop placed within the sampling area. Neither the thermal camera nor the laptop generated considerable heat or had fan components that might influence turbulence within the sampling area.

The capture of a thermal images during sampling continued until the camera ran out of battery or the camera-PC connection was lost (which could be identified by sequential duplicate images that did not show robot motion which should have been visible). Due to constraints of the software, loss of the PC connection often occurred before the end of floral sampling. Thermal images captured more than 151 min after the start of sampling were also discarded as sampling had finished by this point. All remaining thermal images were included in our analysis of floral temperature over the sampling period, regardless of when camera connection was lost.

The temperature of samples was taken from the thermal images in FLIR tools using a manually placed point measurement at the centre of the visible portion of each flower or control tube. During thermographic measurements, target emissivity was set to 0.98, a value appropriate for floral and vegetative tissue (Harrap and Rands 2021a), and reflected temperature measured using the multidirectional mirror placed in frame (Harrap et al. 2018): distance was assumed to be 1 m, while humidity and environmental temperature, which have only minor effects on measurements (Usamentiaga et al. 2014; Vollmer and Möllmann 2017), were set to 50% and 20 °C, respectively. Application of gel, being composed of different material to plant tissue and differing in texture, may alter object emissivity. Although similar Vaseline mixtures have been found to lower the infrared emissivity of targets like skin, this effect is very small (Steketee 1976) and previous thermography work measuring Vaseline-treated leaves with thermographic tools deemed it unnecessary to change emissivity settings between treated and untreated leaves (Jones 1999; Leinonen and Jones 2004; Grant et al. 2006; Stoll and Jones 2007). Consequently, we chose to not change emissivity for gel-covered targets.

As different treatments were applied to flower species (‘Unhandled’, ‘Untreated’, and ‘Gel’), and the Dry Tube controls (‘Untreated’ and ‘Gel’), the temperatures of each flower species and the Dry tube controls were analysed separately to avoid rank deficiencies and avoiding complex three factor interactions. For both species and the Dry Tube controls, the effects of treatment and time elapsed during sampling (measured as decimalized minutes) were assessed using a repeated-measures ANOVA, including both flower or control identity and the pairings of samples (i.e., which samples were monitored at the same time) as a random factors.

## Results

### Control samples

**Fig. 4:**
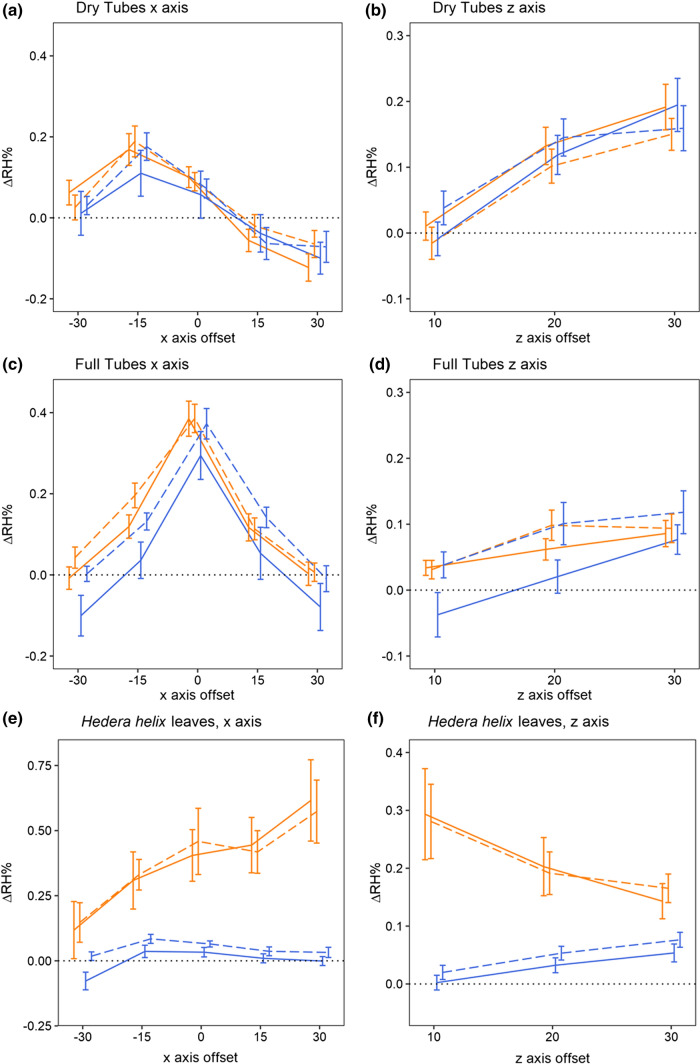
The effect of antitranspirant gel treatments on the humidity production of tube controls and leaf samples. Plots show mean difference in humidity relative to the background (ΔRH) across the *x* (**a**, **c**, **e**) and *z* (**b**, **d**, **f**) axis transects of Dry tube controls (**a**, **b**), Full tube controls (**c**, **d**), and *Hedera helix* leaves (**e**, **f**). All axis offsets are relative to the transect central point and in millimetres. The thin dotted line indicates a 0% change in humidity (the background level). Bold lines plot the mean ΔRH of each treatment of each sample at each replicate transect. Error bars represent ± SE. *n* values for each treatment of each tube control and leaf samples are given in detail in Table 1. Colour indicates treatment: orange the ‘Untreated’ treatment; blue the ‘Gel’ treatment. Dashing of lines indicates the transect replicate: solid, first transect; dashed, the second transect. Note that positioning of lines and bars is offset from the measurement point in the *x-* and *z*-axis for clarity

In the Dry Tube control, we saw little change in the humidity during sampling (Fig. 4a and b, Table 2). Best-fitting models, according to AIC (Table 2), indicated that application of gel had no influence on humidity production in either *x* or *z* transects. This led to models without treatment effects (*T0* and *Tz0*) being favoured, which indicates that the presence of gel on its own did not produce or reduce the humidity of Dry Tube controls.

**Table 2:** Summary of humidity and the influences of treatments on control samples, *Hedra helix* leaves and flowers (hereafter ‘samples’)

Sample	*x*-axis model		*z*-axis model		Treatment	${X}_{t}^{\mathrm{max}}$ (mm)	${\Delta RH}_{x}^{\mathrm{max}}$ (%)
	Struc	Treat	Struc	Treat			
Dry tube control	*m3*_*Q*_	*T0*	*z1*_*L*_	*Tz0*	Untreated	− 12.1	0.10
					Gel-treated	− 12.1	0.10
Full tube control	*m6*_*Q*_	*T1(T2)*	*z3*_*L*_	*Tz0*	Untreated	0	1) 0.25
							2) 0.31
					Gel-treated	0	1) 0.20
							2) 0.26
*Hedera helix* leaves	*m1*_*L*_	*T3*	*z1*_*L*_	*Tz3*	Untreated	30	0.59
					Gel-treated	30	0.03
*Calystegia silvatica* flowers	*m7*_*Q*_	*T4*	*z3*_*L*_	*Tz3*_*TCwU*_	Unhandled	5.80	1) 0.92
							2) 0.82
					Untreated	3.88	1) 1.26
							2) 1.10
					Gel-treated	1.59	1) 0.35
							2) 0.35
*Eschscholzia californica* flowers	*m3*_*Q*_	*T3*	*z1*_*L*_	*Tz3*_*TCwU*_	Unhandled	3.44	0.97
					Untreated	3.34	1.33
					Gel-treated	2.22	0.64

For each transect (*x* and *z*) of each sample, the best-fitting model of humidity structure (column ‘struc.’) and treatment effects (column ‘treat.’) are given. Where more than one comparable models are best both are given, model outside bracket indicates the lower AIC model used for subsequent calculations. Subscript letters following ‘struc.’ models indicate the shape of humidity described by the best-fitting model: ‘*L*’ a linear (*x*-axis) or log-linear (*z*-axis) structure, and ‘*Q*’ a quadratic structure (*x*-axis only). For each treatment of each sample, ${X}_{t}^{\mathrm{max}}$ and ${\Delta RH}_{x}^{\mathrm{max}}$ values estimated by the best-fitting *x-*axis ‘treat.’ model a given. Changes in humidity structure and intensity between treatments are indicated by corresponding differences in ${X}_{t}^{\mathrm{max}}$ and ${\Delta RH}_{x}^{\mathrm{max}}$, respectively. Where the best model of a sample indicates a change in ${\Delta RH}_{x}^{\mathrm{max}}$ with replicate transects ${\Delta RH}_{x}^{\mathrm{max}}$ of both are given: ‘1)’ indicates the first transects; ‘2)’ the second. For further detail on model identity and structure, see Supplementary Information S2. For expanded results of AIC tests for each sample type, see Supplementary Information S4

When horticultural tubes were filled with water (the Full Tube control), humidity production increased, as seen by the slightly elevated ${\Delta RH}_{x}^{\mathrm{max}}$ values (Table 2). According to the best-fitting humidity structure model, humidity production showed a quadratic structure with peak humidity at the centre of the transect (Fig. 4c), indicating that the source of this humidity was evaporation of water through the hole in tube lids. A small rise in humidity with replicate transects suggests that this evaporation slowly accumulated over the sampling period after tubes were moved to their position in the sampling area. Application of gel to the Full Tube control influenced humidity production slightly. Models that allowed gel treatment to either cause a 0.05% drop in relative humidity (*T1* treatment model) or a small change in humidity structure (*T2* treatment model) performed best. However, these models were comparable in terms of AIC (Table 2, Supplementary Information S4).

In the *z*-axis transect, both control samples showed a (log-)linear structure according to the best-fitting humidity structure model (Fig. 4b and d). Humidity production, $\Delta RH$, nearer the control samples (*z* offset = 10 mm) was approximately 0%, with humidity rising by small amounts (*c*.0.1–0.2%) with increased distance. Treatments had no effect on humidity production in the *z*-axis transect in either control, leading to models with no treatment effects (*Tz0* models) being favoured in both control samples (Table 2). Best-fitting models of the Full Tube control suggest that extraneous humidity sources were low (${\Delta RH}_{x}^{\mathrm{max}}$ was between 0.25 and 0.31%, that of the Untreated Full tube control).

### Leaf samples

‘Untreated’ *H. helix* leaf transects found humidity about the leaf to be elevated by a small amount (${\Delta RH}_{x}^{max}$ = 0.59%). Humidity produced by untreated leaves increased across the *x*-axis, and best-fitting models of leaf *x*-axis transects indicated a linear humidity structure of untreated leaves (Fig. 4e, Table 2). In *z*-axis transects, humidity declined with increased distance from untreated leaves (Fig. 4f, Table 2).

Gel application on *H. helix* leaves changed both the structure and amount of humidity produced by leaf samples, resulting in *T3* and *Tz3* treatment models being favoured (Fig. 4e and f, Table 2). Gel treatment reduced the amount of humidity produced by leaves in the *x*-axis almost completely (${\Delta RH}_{x}^{\mathrm{max}}$ = 0.03%). This was paired with a flattening in humidity structure over the *x*-axis transect, meaning that $\Delta RH$ remained at near zero levels across the *x* transects of treated leaves (Fig. 4e). In the *z* transect, humidity intensity was likewise reduced, but structure changed, so that humidity rose slightly over the *z* transect (Fig. 4f), as in control samples.

### Flower samples

**Fig. 5:**
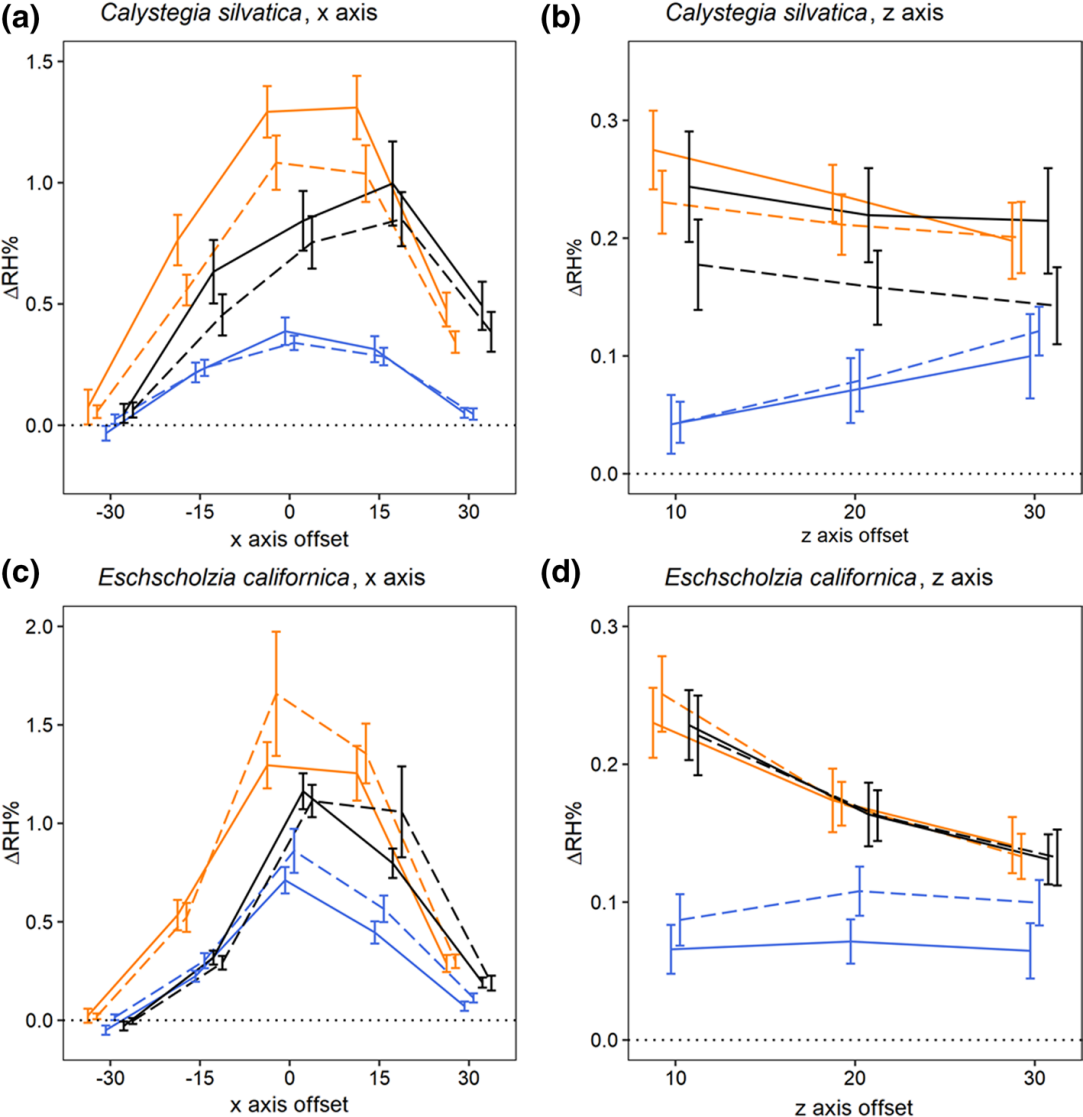
The effect of antitranspirant gel treatments on the humidity production of flowers. Plots show mean difference in humidity relative to the background (ΔRH) across the *x* (**a**, **c**) and *z* (**b**, **d**) axis transects of *Calystegia silvatica* (**a**, **b**), and *Eschscholzia californica* (**c**, **d**). Details as for Fig. 4, noting that colour here indicates treatment: black, the ‘Unhandled’ treatment; orange, the ‘Untreated’ treatment; blue, the ‘Gel’ treatment. *n* values for each treatment of each flower species are given in detail in Table 1

Floral humidity detected in the headspaces of ‘Unhandled’ *C. silvatica* and *E. californica* flowers (Fig. 5) was broadly consistent with floral humidity observed previously (Harrap et al. 2020a). Best-fitting models indicated that floral humidity in both species showed a quadratic structure across the *x*-axis transect (Fig. 5a and c, Table 2). In both species, ${\Delta RH}_{x}^{max}$ was close to 1% in Unhandled flowers. In the *z*-axis transect, both species slowed a decrease in humidity with increased distance from the flower (Fig. 5b and d). Furthermore, best-fitting models indicated that *C. silvatica* floral humidity decreased slightly with replicate transects in the x-axis. In *E. californica*, humidity was not found to differ between replicate transects.

The treatments influenced both the intensity and structure of floral humidity in both transects of both species (Fig. 5, Table 2), and influenced how *C. silvatica* humidity intensity changed with replicate transects within the *x*-axis. For the *x-* and *z*-axis transects, respectively, the *T3* and *Tz3*_*TCwU*_ treatment effect models were favoured by AIC for *E. californica,* and the *T4* and *Tz3*_*TCwU*_ models for *C. silvatica* (Table 2).

Handling flowers as if gel were being applied (the ‘Untreated’ treatment) led to a small increase in humidity production compared to flowers of the same species that had not been handled (the ‘Unhandled’ treatment, Fig. 5). This increase in humidity production was similar between both species with ${\Delta RH}_{x}^{max}$ increasing in relative humidity in ‘Untreated’ flowers compared to ‘Unhandled’ flowers by 0.36% in *E. californica* and 0.34% in the initial transect of *C. silvatica*, 0.28% in the second transect. In *z*-axis transects of both species, humidity was found not to differ between ‘Unhandled’ and ‘Untreated’ treatments (Table 2).

In both *C. silvatica* and *E. californica*, application of gel to petal surfaces to block transpiration (the ‘Gel’ treatment) reduced floral humidity production compared to other treatments (Fig. 5). Humidity intensity of ‘Gel’ treated *C. silvatica* flowers was approximately a third of that in ‘Untreated’ flowers (${\Delta RH}_{x}^{\mathrm{max}}$ being 0.35% in both transects). In *E. californica*, floral humidity intensity of ‘Gel’ treated flowers was approximately half that of ‘Untreated’ flowers (${\Delta RH}_{x}^{\mathrm{max}}$ being 0.64%). These decreases were accompanied by a flattening of the *x*-axis humidity structure in both species (Fig. 5). Additionally, in *C. silvatica* floral humidity production of gel-treated flowers was more consistent, remaining at a reduced level between replicate transects. In the *z*-axis transects, humidity was likewise reduced by gel treatments in both species, but in *C. silvatica* humidity was further affected with *z*-axis humidity structure changing, so that humidity rose slightly over the *z* transect (Fig. 5), much like that of Dry Tube control samples.

### Treatments and temperature of flowers and controls

Temperature monitoring of Dry Tube controls during humidity sampling revealed application of gel had no detectable effect on tube surface temperature (*min elapsed and gel treatment interaction effects* ANOVA, *F*_1,37_ = 0.438, *P* = 0.512; *gel treatment* ANOVA, *F*_1,37_ = 0.273 *P* = 0.605). Furthermore, tubes did not show a change in temperature over time (*min elapsed* ANOVA, *F*_1,37_ = 0.502, *P* = 0.483). That gel treated tubes did not differ in temperature suggests the gel itself does not generate heat. Gel treatment likewise had no effect on floral surface temperature of *E. californica* flowers (*min elapsed and gel treatment interaction effects* ANOVA, *F*_2,148_ = 1.023, *P* = 0.362; *gel treatment* ANOVA, *F*_2,14_ = 1.048 *P* = 0.376). *E. californica* flowers began sampling at approximately the temperature of the sampling room (22.58 °C, according to ANOVA model mean estimates) and cooled slightly (at a rate of − 8.5 × 10^–4^ °C/min) during humidity sampling (*min elapsed* ANOVA, *F*_1,149_ = 4.716, *P* = 0.031). However, gel treatment did affect the temperature of *C. silvatica* flowers, which showed a similar change in temperature with time regardless of treatment (*min elapsed and gel treatment interaction effects* ANOVA, *F*_2,176_ = 1.287*, P* = 0.279), with flowers cooling gradually (at a rate of − 6.1 × 10^–4^ °C/min) over time (*min elapsed* ANOVA, *F*_1,176_ = 13.889, *P* < 0.001). Nevertheless, gel treatment had a small but significant effect on the overall temperature of the flowers (*gel treatment* ANOVA, *F*_2,16_ = 6.753, *P* = 0.008). Post hoc Tukey tests revealed that this was due to ‘Gel’ treated flowers being, on average, 0.3 °C hotter than ‘Untreated’ flowers (*Untreated-Gel comparison*, Tukey test, *P* = 0.025), ‘Untreated’ flowers again beginning sampling at approximately room temperature (22.53 °C, according to ANOVA model mean estimates). No significant temperature differences were found between other treatment group pairings of *C. silvatica* flowers (*Untreated-Unhandled comparison*, Tukey test, *P* = 0.231; *Gel-Unhandled comparison*, Tukey test, *P* = 0.996).

## Discussion

Antitranspirant treatment of flower petals and sepals and the resulting effects on floral humidity confirm that transpiration contributes to the generation of floral humidity. Gel-treated flowers produced less floral humidity than flowers that received no gel treatments (Fig. 5, Table 2). This gel antitranspirant did not produce or absorb water vapour itself, confirmed by the lack of treatment effects on Dry Tube control samples (Fig. 4a and b). The gel treatment did influence the sample humidity of the Full Tube control sample, where humidity was reduced very slightly (Fig. 4c and d). Although, as gel treatment did not change humidity production in the Dry Tube control, this small influence of the gel treatment on the Full Tube Control is likely due to gel slightly blocking evaporation of water within the tube, pooling in such a way that it obscures or effectively narrows the hole in the tube’s lid. A similar small effect of obscuring the hole in the tubes lid was observed in Harrap et al. (2020a). Furthermore, as *H. helix* leaf humidity generation would be predominantly through transpiration (Vezza et al. 2006; von Arx et al. 2012), the almost complete removal of leaf headspace humidity with gel treatment confirmed that the gel treatment effectively blocks transpiration from treated plant tissues and consequently removes the contribution of transpiration to sample headspace humidity (Fig. 4d and e). Handling during gel application affected humidity, evidenced by the slightly increased floral humidity of ‘Untreated’ flowers of both species compared to ‘Unhandled’ flowers (Table 2). Such changes in humidity production due to handling alone may be due to micro-abrasions and cell damage to petal surfaces during handling, or transfer of grease or moisture from fingers. Regardless, this conflating handling effect was small. Considering the results together, the reduction in humidity production of flowers with gel treatments appears to represent the effect of gel removing the contribution of transpiration from floral humidity production. Thus, the results of floral treatments confirm previous, but until now untested, inferences that floral humidity is produced in part by floral transpiration (von Arx et al. 2012; Harrap et al. 2020a).

The gel did not itself generate heat, as temperatures of Dry tube controls were unaffected by treatment. Likewise, temperature of *E. californica* flowers was not influenced by gel treatments. However, gel treatment did slightly increase *C. silvatica* floral temperature. That ‘Untreated’ flowers were either the same temperature as, or slightly cooler than, gel-treated flowers confirm that floral humidity seen in ‘Untreated’ flowers is not the result of elevated temperatures enhancing nectar evaporation, or transpiration, relative to gel-treated flowers. The rise in temperature with gel treatment *C. silvatica* appears due to the gel blocking transpiration, compromising the flowers’ capacity to lose heat, observed with similar antitranspirant treatments of other Convolvulaceae (Patiño and Grace 2002). This effect may be greater in *C. silvatica* due to its larger size, limiting passive heat loss with air conduction, explaining why temperature differences were not detected in *E. californica*. That preventing transpiration impacts both temperature and humidity in *C. silvatica* provides evidence that a relationship between floral humidity and floral temperature exists though the influence of floral transpiration. That only small increases in temperature were created when heat loss from transpiration is removed suggests that flowers were not under much heat stress during sampling; thus, only small amounts of heat accumulate by preventing transpirational heat loss.

Our results indicate that transpiration can have quite large contributions to floral humidity generation and species can vary in the extent that floral humidity is produced by transpiration. Although total floral humidity production in Untreated flowers was similar in both species, transpiration was found to have a greater contribution to floral humidity production in *C. silvatica*, accounting for a larger part of the total floral humidity produced than in *E. californica*. In *C. silvatica*, it appears transpiration has a greater contribution relative to other sources. The differences between gel-treated flowers and flowers in other treatments indicates transpiration accounts for about a half of the total floral humidity produced by *E. californica*, and two-thirds of that produced by *C. silvatica*. However, floral transpiration may have a greater contribution to total floral humidity and then indicated by our treatments. Neither floral reproductive structures nor areas proximal to them (including nectaries) were gel treated. These structures may also transpire and this may contribute to the remaining humidity production of gel-treated flowers. Additionally, the elevated humidity detected in the floral headspace of gel-treated flowers still includes humidity from sources extraneous to the flower. These ‘extraneous sources’ may account for differences between focal and background probes of up to 0.31% relative humidity (that of the ‘Untreated’ full tube). However, leaf and flower samples also block the hole in the horticultural tube lid. Consequently, extraneous humidity sources are likely to be lowered in a similar manner as seen in Harrap et al. (2020a) and the ‘Gel’ treated Full Tube control.

Several influences determine the capacity of a flower to generate floral humidity (Harrap et al. 2020a). These include evaporation of nectar, which produces humidity, and floral structure, which influences how it accumulates in flower headspaces (von Arx et al. 2012). Additionally, environmental factors, particularly air temperature, will influence nectar evaporation rates. Environmental factors may also affect how humidity is allowed to accumulate. Movement of air from wind may lead to weather-dependent disruption of floral humidity by carrying vapour away from the flower headspace; in the same way, wind disrupts floral scent plumes (Lawson et al. 2017), and disruption of boundary layers about the flower. Such wind disruptions may be moderated by floral architecture, with less open floral shapes creating shielded environments for floral humidity to accumulate (Corbet et al. 1979a, b; Harrap et al. 2020a). Our results confirm, as previously inferred, that transpiration also contributes to the amount of floral humidity generated. This means floral traits and environmental conditions that influence floral transpiration will impact the capacity of flower species to produce floral humidity, and differences in these floral transpiration traits may help explain the diversity of floral humidity produced across the angiosperms (Harrap et al. 2020a).

### *Author contribution statement*

SAR provided resources, supervision, project administration, and acquired funding. MJMH developed the methodology for this study, conducted the investigation, data curation, and visualisation, and conducted formal analysis. Both authors were involved in study conceptualization and together wrote the manuscript.

## Supplementary Information

Below is the link to the electronic supplementary material.

Supplementary file1 (DOCX 85 KB)

## Data Availability

All of the datasets generated during and analysed during the current study are available in the Figshare repository, https://doi.org/10.6084/m9.figshare.14350547 (Harrap and Rands 2021b).

## Abbreviation

AIC: Akaike information criterion

## References

[CR1] Ashman T-L, Knight TM, Steets JA (2004). Pollen limitation of plant reproduction: ecological and evolutionary causes and consequences. Ecology.

[CR2] Azad AK, Sawa Y, Ishikawa T, Shibata H (2007). Temperature-dependent stomatal movement in tulip petals controls water transpiration during flower opening and closing. Ann Appl Biol.

[CR3] Baude M, Kunin WE, Boatman ND (2016). Historical nectar assessment reveals the fall and rise of floral resources in Britain. Nature.

[CR4] Buschhaus C, Hager D, Jetter R (2015). Wax layers on *Cosmos bipinnatus* petals contribute unequally to total petal water resistance. Plant Physiol.

[CR5] Cheng G, Huang H, Zhou L (2019). Chemical composition and water permeability of the cuticular wax barrier in rose leaf and petal: a comparative investigation. Plant Physiol Biochem.

[CR6] Clarke D, Whitney H, Sutton G, Robert D (2013). Detection and learning of floral electric fields by bumblebees. Science.

[CR7] Corbet SA, Unwin DM, Prŷs-Jones OE (1979). Humidity, nectar and insect visits to flowers, with special reference to *Crataegus*, *Tilia* and *Echium*. Ecol Entomol.

[CR8] Corbet SA, Willmer PG, Beament JWL (1979). Post-secretory determinants of sugar concentration in nectar. Plant Cell Environ.

[CR9] Davenport DC, Fisher MA, Hagan RM (1972). Some counteractive effects of antitranspirants. Plant Physiol.

[CR10] Dyer AG, Chittka L (2004). Fine colour discrimination requires differential conditioning in bumblebees. Naturwissenschaften.

[CR11] Dyer AG, Whitney HM, Arnold SEJ (2006). Bees associate warmth with floral colour. Nature.

[CR13] Foster JJ, Sharkey CR, Gaworska AVA (2014). Bumblebees learn polarization patterns. Curr Biol.

[CR14] Gates DM (1968). Transpiration and leaf temperature. Annu Rev Plant Physiol.

[CR15] Grant OM, Chaves MM, Jones HG (2006). Optimizing thermal imaging as a technique for detecting stomatal closure induced by drought stress under greenhouse conditions. Physiol Plant.

[CR16] Guo Y, Busta L, Jetter R (2017). Cuticular wax coverage and composition differ among organs of *Taraxacum officinale*. Plant Physiol Biochem.

[CR17] Hajibagheri MA, Hall JL, Flowers TJ (1983). The structure of the cuticle in relation to cuticular transpiration in leaves of the halophyte *Suaeda maritima* (L.) Dum. New Phytol.

[CR18] Harrap MJM, Rands SA (2021). Floral infrared emissivity estimates using simple tools. Plant Methods.

[CR19] Harrap MJM, Rands SA (2021). Figshare Database.

[CR20] Harrap MJM, Rands SA, Hempel de Ibarra N, Whitney HM (2017). The diversity of floral temperature patterns, and their use by pollinators. Elife.

[CR21] Harrap MJM, Hempel de Ibarra N, Whitney HM, Rands SA (2018). Reporting of thermography parameters in biology: a systematic review of thermal imaging literature. R Soc Open Sci.

[CR22] Harrap MJM, Hempel de Ibarra N, Knowles HD (2020). Floral humidity in flowering plants: a preliminary survey. Front Plant Sci.

[CR23] Harrap MJM, Hempel de Ibarra N, Whitney HM, Rands SA (2020). Floral temperature patterns can function as floral guides. Arthropod-Plant Interact.

[CR24] Harrap MJM, Hempel de Ibarra N, Knowles HD (2021). Bumblebees can detect floral humidity. J Exp Biol.

[CR25] Hase AV, Cowling RM, Ellis AG (2006). Petal movement in cape wildflowers protects pollen from exposure to moisture. Plant Ecol.

[CR26] Hew CS, Lee GL, Wong SC (1980). Occurrence of non-functional stomata in the flowers of tropical orchids. Ann Bot.

[CR27] Hicks DM, Ouvrard P, Baldock KCR (2016). Food for pollinators: quantifying the nectar and pollen resources of urban flower meadows. PLoS ONE.

[CR28] Huang X, Lin S, He S (2018). Characterization of stomata on floral organs and scapes of cut ‘Real’ gerberas and their involvement in postharvest water loss. Postharvest Biol Technol.

[CR29] Jolliet O, Bailey BJ (1992). The effect of climate on tomato transpiration in greenhouses: measurements and models comparison. Agric for Meteorol.

[CR30] Jones HG (1999). Use of thermography for quantitative studies of spatial and temporal variation of stomatal conductance over leaf surfaces. Plant Cell Environ.

[CR31] Kunze J, Gumbert A (2001). The combined effect of color and odor on flower choice behavior of bumble bees in flower mimicry systems. Behav Ecol.

[CR32] Lawson DA, Whitney HM, Rands SA (2017). Colour as a backup for scent in the presence of olfactory noise: testing the efficacy backup hypothesis using bumblebees (*Bombus terrestris*). R Soc Open Sci.

[CR33] Lawson DA, Chittka L, Whitney HM, Rands SA (2018). Bumblebees distinguish floral scent patterns, and can transfer these to corresponding visual patterns. Proc R Soc B.

[CR34] Leinonen I, Jones HG (2004). Combining thermal and visible imagery for estimating canopy temperature and identifying plant stress. J Exp Bot.

[CR35] Leonard AS, Masek P (2014). Multisensory integration of colors and scents: insights from bees and flowers. J Comp Physiol A.

[CR36] Leonard AS, Dornhaus A, Papaj DR (2011). Forget-me-not: complex floral displays, inter-signal interactions, and pollinator cognition. Curr Zool.

[CR37] Leonard AS, Dornhaus A, Papaj DR, Patiny S (2012). Why are floral signals complex? An outline of functional hypotheses. Evolution of plant–pollinator relationships.

[CR38] Lü P, Huang X, Li H (2011). Continuous automatic measurement of water uptake and water loss of cut flower stems. HortScience.

[CR40] Muth F, Papaj DR, Leonard AS (2015). Colour learning when foraging for nectar and pollen: bees learn two colours at once. Biol Lett.

[CR41] Nepi M, Franchi GG, Padni E (2001). Pollen hydration status at dispersal: cytophysiological features and strategies. Protoplasma.

[CR42] Neumann PM (1974). Senescence of attached bean leaves accelerated by sprays of silicone oil antitranspirants. Plant Physiol.

[CR43] Nordström K, Dahlbom J, Pragadheesh VS (2017). In situ modeling of multimodal floral cues attracting wild pollinators across environments. Proc Natl Acad Sci USA.

[CR44] Patiño S, Grace J (2002). The cooling of convolvulaceous flowers in a tropical environment. Plant Cell Environ.

[CR45] Raguso RA (2004). Flowers as sensory billboards: progress towards an integrated understanding of floral advertisement. Curr Opin Plant Biol.

[CR46] Raine NE, Chittka L (2008). The correlation of learning speed and natural foraging success in bumble-bees. Proc R Soc B.

[CR47] Rawson HM, Begg JE, Woodward RG (1977). The effect of atmospheric humidity on photosynthesis, transpiration and water use efficiency of leaves of several plant species. Planta.

[CR48] Richards SA (2008). Dealing with overdispersed count data in applied ecology. J Appl Ecol.

[CR49] Schiestl FP, Johnson SD (2013). Pollinator-mediated evolution of floral signals. Trend Ecol Evol.

[CR50] Schreiber L (2001). Effect of temperature on cuticular transpiration of isolated cuticular membranes and leaf discs. J Exp Biol.

[CR51] Simon NML, Graham CA, Comben NE (2020). The circadian clock influences the long-term water use efficiency of Arabidopsis. Plant Physiol.

[CR52] Steketee J (1976). The influence of cosmetics and ointments on the spectral emissivity of skin (skin temperature measurement). Phys Med Biol.

[CR53] Stoll M, Jones HG (2007). Thermal imaging as a viable tool for monitoring plant stress. OENO One.

[CR54] Tichy H, Kallina W (2014). Sensitivity of honeybee hygroreceptors to slow humidity changes and temporal humidity variation detected in high resolution by mobile measurements. PLoS ONE.

[CR55] Usamentiaga R, Venegas P, Guerediaga J (2014). Infrared thermography for temperature measurement and non-destructive testing. Sensors.

[CR56] van Doorn WG (1997). Water relations of cut flowers. Hortic Rev.

[CR57] Vezza M, Nepi M, Guarnieri M (2006). Ivy (*Hedera helix* L.) flower nectar and nectary ecophysiology. Inter J Plant Sci.

[CR58] Vollmer M, Möllmann K-P (2017). Infrared thermal imaging: fundamentals, research and applications.

[CR59] von Arx M (2013). Floral humidity and other indicators of energy rewards in pollination biology. Commun Integr.

[CR60] von Arx M, Goyret J, Davidowitz G, Raguso RA (2012). Floral humidity as a reliable sensory cue for profitability assessment by nectar-foraging hawkmoths. Proc Natl Acad Sci USA.

[CR61] Whitney HM, Dyer AG, Chittka L (2008). The interaction of temperature and sucrose concentration on foraging preferences in bumblebees. Naturwissenschaften.

[CR62] Whitney HM, Chittka L, Bruce TJA, Glover BJ (2009). Conical epidermal cells allow bees to grip flowers and increase foraging efficiency. Curr Biol.

[CR63] Whitney HM, Bennett KMV, Dorling M (2011). Why do so many petals have conical epidermal cells?. Ann Bot.

[CR64] Wolfin MS, Raguso RA, Davidowitz G, Goyret J (2018). Context dependency of in-flight responses by *Manduca sexta* moths to ambient differences in relative humidity. J Exp Biol.

[CR65] Wright GA, Schiestl FP (2009). The evolution of floral scent: the influence of olfactory learning by insect pollinators on the honest signalling of floral rewards. Funct Ecol.

